# Neurologists’ insights and practices on generic antiepileptic medications in epilepsy management: A Saudi Arabian perspective

**DOI:** 10.12669/pjms.41.7.11981

**Published:** 2025-07

**Authors:** Bandar Nasser Aljafen, Reem Nafel Alqahtani, Reem Saeed Algarni, Hend Elmutawi, Mohamed Hamdy Bahr

**Affiliations:** 1 Bandar Nasser Aljafen Department of Medicine (Neurology Unit), College of Medicine, King Saud University, Riyadh, Saudi Arabia; 2 Reem Nafel Alqahtani Department of Adult Neurology, King Faisal Specialist Hospital and Research Centre (KFSHRC), Riyadh, Saudi Arabia; 3 Reem Saeed Algarni Department of Family Medicine, King Saud Medical City, Riyadh, Saudi Arabia; 4 Hend Elmutawi College of Medicine, King Saud University, Riyadh, Saudi Arabia; 5 Mohamed Hamdy Bahr Department of Clinical Pharmacology, Faculty of Medicine, Ain Shams University, Cairo, Egypt, and Department of Basic Medical Sciences, Vision Colleges, Riyadh, Saudi Arabia

**Keywords:** Epilepsy, Neuro-physicians, Perception, Antiepileptic medications, Saudi Arabia

## Abstract

**Objectives:**

This study aimed to investigate neurologists’ perceptions and practices regarding generic antiepileptic medications (AEDs) in the management of epilepsy, and whether generic AEDs can be used as a satisfactory, effective, and safe substitute for brand-name medications in targeted patients.

**Methods:**

This questionnaire based cross-sectional study was performed in the Department of Medicine (Neurology Division), College of Medicine, King Saud University, Riyadh, Saudi Arabia, from July 2022 to July 2024. One hundred thirty neuro physicians participated, and 114 completed the questionnaire, yielding a response rate of 87.7%. The participants consisted of 63.2% males and 36.8% females. The questions were based on a 3-point Likert scale.

**Results:**

The results revealed that all four components were strongly and positively correlated with each other: the ‘Patient Miscontrol with Generic AEDs’ component had a statistically significant strong positive correlation with ‘concern About Generic AEDs’ component, r(114) = 0.654, p < 0.0005; with ‘Aversion to Generic AEDs’ component, r(114) = 0.525, p < 0.0005; and ‘Withholding Generic AEDs’ component, r(114) = 0.595, p < 0.0005. ‘Concern About Generic AEDs’ component had a statistically significant strong positive correlation with the ‘Aversion to Generic AEDs’ component, r (114) = 0.538, p < 0.0005; and the ‘Withholding Generic AEDs’ component, r (114) = 0.459, p < 0.0005. Finally, the ‘Aversion to Generic AEDs’ component showed a statistically significant positive correlation with the ‘Withholding Generic AEDs’ component, r (114) = 0.435, p < 0.0005. Spearman correlation analysis revealed a positive correlation between years of experience and ‘Withholding Generic AEDs;’ r (114) = 0.243, p = 0.009. The post hoc analysis revealed statistically significant differences between the low-experienced ‘< 5 years’ (66.50) and the high-experienced ‘> 10 years’ (100.00) groups (p = 0.014).

**Conclusions:**

Half of the neurologists participating in the study are concerned about substituting brand-name antiepileptic drugs (AEDs) with generic ones. Neurologists had mixed perceptions regarding the use of generic AEDs for cost-saving purposes and the safety of substituting brand-name medications with generics. We encourage reporting every incident of seizure in patients on generic AEDs.

## INTRODUCTION

Epilepsy is a chronic, noncommunicable neurological disorder that is one of the most common globally. As per the World Health Organization report 2024^1^, about 50 million people worldwide have epilepsy, 80% from low-middle-income countries. People with epilepsy have a mortality risk that is approximately three times higher than that of the general population. The incidence of epilepsy is mainly in low-income countries.^1^,^2^ Epilepsy impacts individuals across all age groups, contributing to significant morbidity and mortality, and it imposes a substantial burden on patients’ daily lives and the healthcare system.^3^

Most patients need long-term treatment with antiepileptic drugs (AEDs). Meanwhile, generic AEDs offer a cost-effective alternative to reduce treatment expenses.^4^ Generic medication provides an efficient solution as it mimics the drug’s efficacy with an up to 85% reduction in cost.^5^ However, substituting brand-name AEDs with generic versions and switching among generic AED products is a controversial practice. Physicians and patients often express concerns about increased seizure frequency and adverse events while switching from brand-name to generic drugs.^2^ Hence, switching patients with controlled epilepsy to a generic AED has to be done under a cautious approach; otherwise, this might create another dilemma and significantly increase the burden of epilepsy as the prevalence of multi-drug resistance epilepsy in Saudi Arabia is already estimated to be about 30%.^6^

The present study aimed to investigate the perception and practice of neuro-physicians towards generic antiepileptic medications in the management of epilepsy and whether generic AEDs can satisfactorily be used as a natural, effective, and safe substitute for brand-name medications in such patients.

## METHODS

This questionnaire-based cross-sectional study was conducted in the Department of Medicine (Neurology Division) at King Saud University’s College of Medicine, Riyadh, Saudi Arabia, from July 2022 to July 2024.

### Questionnaire:

The research team prepared a well-established English-language questionnaire, which two faculty members reviewed. The questionnaire was based on the study Haskins et al.^7^ and permission was obtained. The questionnaire was initially piloted among six faculty members to identify questions that might not be clear to the participants and possibly need modification. After that, the questionnaire was distributed via social media platforms, including WhatsApp and email, to collect the data. The targeted population was adult and pediatric neurologists registered in the Saudi Commission for Health Specialties (SCHS), who were contacted and received the survey through email and social media contact information.

### Inclusion and exclusion criteria:

This study included all adult and pediatric-certified neurologists working in Saudi Arabia. Physicians without certified neurologist status and neurologists working outside Saudi Arabia were excluded.

### Ethical Consideration and Statistical Analysis:

The Institutional Review Board (IRB), College of Medicine, King Saud University, approved the project (E-22- E-22-6976; dated: July 17, 2022). A consent statement was attached to the questionnaire, indicating the study’s purpose and the participant’s right to withdraw without any obligation from the study team. Descriptive statistics (percentages, means, and standard deviations) were used to identify the demographic and other characteristics. Principal Component Analysis (PCA) was applied as described in the results section. Correlations among study outcomes were evaluated; Pearson correlation was used to determine the correlation among continuous variables, while Spearman’s correlation was used to determine the correlation when one variable is ordinal. An independent sample *t*-test or one-way ANOVA was conducted to determine whether a significant difference existed between two groups or three or more groups, respectively. Normality was inspected using the Kolmogorov–Smirnov test and inspection of Normal Q-Q plots. Whenever the groups did not pass the normality test, Mann-Whitney U and Kruskal-Wallis tests were conducted to determine whether a significant difference existed between two groups or three or more groups, respectively. For nonparametric tests, comparisons were made in terms of medians. Data are presented as mean ± standard deviation unless otherwise stated. Results were considered significant wherever *p* < 0.05. Data were analyzed using the statistical program SPSS (IBM SPSS) Statistical Package for Social Sciences, for Windows, V23.0, Armonk, NY, USA version 23.0).

## RESULTS

In this study, 130 neurologists were interviewed for the survey. Of these, 114 participants completed the responses; neurologists were from governmental and private hospitals in Riyadh City, constituting an 87.7% response rate. The participants were adult and pediatric neurology specialists. Their age range was 27-70; 63.2% were male, and 36.8% were female. The demographic characteristics of the study participants are shown in Table-I and the clinical practice pattern is shown in Table-II.

**Table-I:** Demographic characteristics of the study participants (n=114).

Characteristic	Participants [Number (Percentage)] (N = 114)
***Age, years***	
21-30	1 (0.9%)
31-40	50 (43.9%)
41-50	32 (28.1%)
51-60	21 (18.4%)
61-70	10 (8.8%)
***Sex***	
Male	72 (63.2%)
Female	42 (36.8%)
***Nationality***	
Saudi	101 (88.6%)
Egyptian	7 (6.1%)
Sudanese	3 (2.6%)
Pakistani	1 (0.9%)
French	1 (0.9%)
German	1 (0.9%)
***Last Certificate Obtained From***	
Saudi Arabia	35 (30.7%)
Egypt	5 (4.4%)
Pakistan	1 (0.9%)
France	1 (0.9%)
Germany	2 (1.8%)
UK	6 (5.3%)
North America	62 (54.4%)
Europe	2 (1.8%)
***Subspecialty***	
Adult Neurologist	64 (56.1%)
Adult Epileptologist	25 (21.9%)
Pediatric Neurologist	15 (13.2%)
Pediatric Epileptologist	10 (8.8%)
***Region of Practice***	
Central Region	55 (48.2%)
South Region	2 (1.8%)
East Region	31 (27.2%)
West Region	26 (22.8%)
***Rural vs. Urban***	
Urban	110 (96.5%)
Rural	4 (3.5%)
***Type of Institute***	
Academic Institute	19 (16.7%)
Secondary Healthcare Center	32 (28.1%)
Tertiary Healthcare Center	63 (55.3%)

**Table-II:** Clinical proficiency or practice of the study participants.

Characteristic	Participants [Number (Percentage)] (N = 114)
***Clinical Experience (Years)***	
<5	15 (13.2%)
5-10	32 (28.1%)
>10	67 (58.8%)
***Number of Clinics (Per Week)***	
2	37 (32.5%)
3	34 (29.8%)
>3	43 (37.7%)
***Number of Patients’ Visits (Per Week)***	
<20	15 (13.2%)
20-40	39 (34.2%)
>40	60 (52.6%)

### Principal Component Analysis:

A principal components analysis (PCA) was run on a 26-questions about neurologists’ perception and practice towards generic AEDs in the management of epilepsy in Saudi Arabia. The questions were on a three point Likert scale. The suitability of PCA was assessed before analysis. Inspection of the correlation matrix showed that all variables had at least one correlation coefficient greater than 0.3. The Kaiser-Meyer-Olkin (KMO) measures show the adequacy of sampling: the overall KMO measure was 0.872 (meritorious’), with individual KMO measures ranging from 0.754-0.914, classifications of ‘middling’ to ‘marvelous’ according to Kaiser (1974). Bartlett’s test of sphericity was statistically significant (*p* < 0.0005), indicating that the data was factorizable.

PCA revealed four components with eigenvalues greater than one and explained 39.97%, 7.66%, 6.92%, and 5.46% of the total variance, respectively. Visual inspection of the scree plot indicated that all four components should be retained (Cattell, 1966). In addition, a four-component solution met the interpretability criterion. As such, four components were retained. The first component includes seven Likert-scale questions, the second entails five, the third entails four, and the fourth entails three questions, as shown in Table-III. Comparisons and correlations were made for component-based scores.

**Table-III:** Rotated component matrix for principal component analysis of questionnaire items.

Questionnaire Item	Component			
	1	2	3	4
24. Substitution with Gen AEDs May ↑ Breakthrough Seizures	0.724			
17. Substituting AEDs Can Have Undesirable Results	0.687			
12. Concerned About Patient Control If Substitution with Gen AEDs	0.662			
14. Concerned About ↑ Breakthrough Seizures After Switch to Gen AEDs	0.623			
22.Permitting/Enforcing Gen AEDs Without Physician Consent is Dangerous (potential impact on patient welfare)	0.590			
25. I Speak with Patients About the Risks/Benefits of Gen AEDs Before Switch	0.581			
19. Substitution of Regular Effective AEDs in Controlled Patients with Gen AEDs May Trigger Breakthrough Seizures	0.566			
6. Concerned About the Safety of Gen AEDs for Short-term		0.861		
3. Concerned About the Safety of Gen AEDs		0.746		
7. Concerned About Efficacy of Gen AEDs for Short-term		0.734		
4. Concerned About Efficacy of Gen AEDs		0.604		
15. Concerned About ↓ Tolerability After Switch to Gen AEDs		0.592		
2. Prefer Gen AEDs for Cost Saving			0.741	
20. I Often Consider Switching Branded AEDs to Gen AEDs			0.662	
8. Uncomfortable Prescribing Gen AEDs			0.657	
16.It Is Safe to Substitute Most Medications with A Generic			0.600	
1.No Substitution in Certain Meds (small diff → AE)				0.792
9. Pharmacist Substitutes with Gen AEDs Physician Consent Only				0.646
13. Believe Universal Substitution with Gen AEDs Without Direct Timely Physician Approval in Epilepsy Inappropriate				0.514

### Extraction method:

Principal Component Analysis. Rotation Method: Varimax with Kaiser Normalization (Rotation converged in six iterations). The four-component solution explained 60.00% of the total variance. A Varimax orthogonal rotation was employed to aid interpretability. The interpretation of the data was consistent with the perception axes the questionnaire was designed to assess, with strong loadings of ‘Generic AEDs decreased Patient Control (Breakthrough Seizures)’ items on Component-1, ‘Physicians Concerned About Generic AEDs’ items on Component-2, ‘Preference decreased for Generic AEDs’ items on Component-3, and ‘Restriction of Dispensing Generic AEDs’ items on Component-4. For simplifying and presentation purposes, we shall designate the components ‘Patient Miscontrol with Generic AEDs,’ Concern About Generic AEDs,’ ‘Aversion to Generic AEDs,’ and ‘Withholding Generic AEDs.’

**Fig.1:**
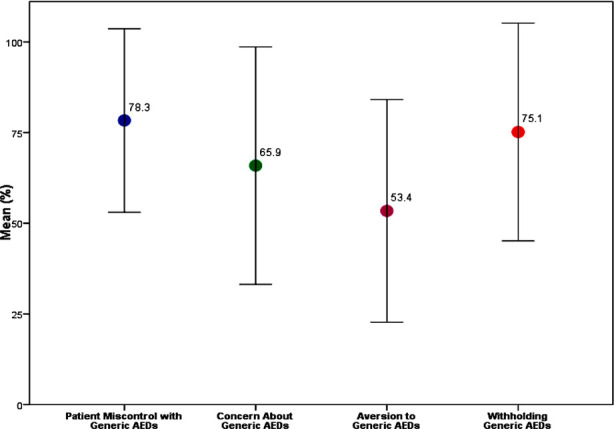
The degree to which participants perceive each component.

The ‘Patient miscontrol with Generic AEDs’ had an average of 78.33 ± 25.31; ‘Concern About Generic AEDs’ had an average of 65.88 ± 32.75, ‘Aversion to Generic AEDs’ had an average of 53.40 ± 30.68, and ‘Withholding Generic AEDs’ had an average of 75.14 ± 30.03. Fig. 1 illustrates the four components and the degree to which participants perceive each component.

### Variance Based on Demographic Attributes:

The Mann-Whitney U and Kruskal-Wallis tests were conducted to determine whether perceptions about Generic AEDs differed, in terms of the four principal components, among participant groups with different genders, specialities, and institutions. For gender comparisons, a Mann-Whitney U test was used. There were 72 male and 42 female participants. Component two median values were statistically significantly higher in females (85.00) than in males (65.00), *U* = 1834, *z* = 1.926, *p* = 0.050. Similarly, component three values were statistically significantly higher in females (75.00) than males (50.00), *U* = 2034.5, z = 3.093, *p* = 0.002.

A Kruskal-Wallis test was conducted to determine if there were differences in the median values between groups that differed in their specialty: the ‘Adult Neurologist,’ Adult Epileptologist,’ Pediatric Neurologist, ’ and ‘Pediatric Epileptologist’. Median values for the ‘Withholding Generic AEDs’ component were statistically significantly different between the different specialities, χ^2^(3) = 13.786, *p* = 0.003. Subsequently, pairwise comparisons were performed using Dunn’s (1964) procedure. A Bonferroni correction for multiple comparisons was made. This post hoc analysis revealed statistically significant differences in the ‘Withholding Generic AEDs’ component between the ‘pediatric neurologist’ (50.00) and the ‘adult neurologist’ (100.00) groups (*p* = 0.002) and ‘pediatric neurologist’ (50.00) and ‘pediatric epileptologist’ (100.00) groups (*p* = 0.020), but not between the ‘adult epileptologist’ group (83.50) or any other group combination.

**Fig.2:**
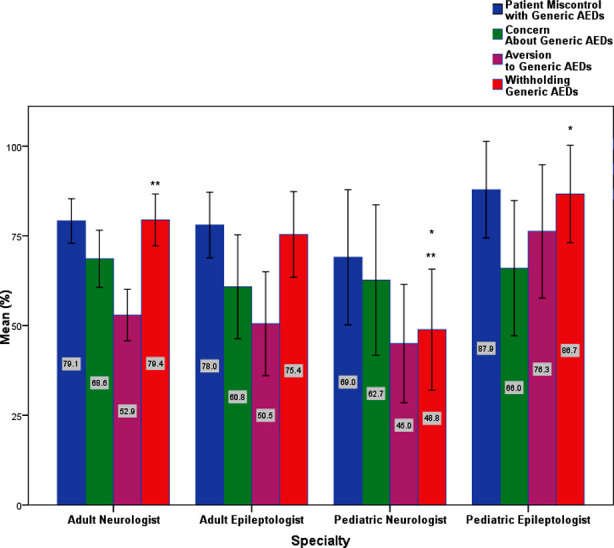
Assessment of neurologists’ perception of using Generic AEDs among different specialities. Bars with asterisks represent group means with significant differences. Error bars represent 95% CI.

Notably, the ‘Aversion to Generic AEDs’ component variance followed a trend towards significance: χ^2^(3) = 6.786, p = 0.073. The post hoc analysis ascribes this difference to the ‘pediatric neurologist’ (50.00) versus the ‘pediatric epileptologist’ (87.5) groups. Fig.2 represents these differences. Fig.2 demonstrates the assessment of neurologists’ perception of using Generic AEDs among different specialities. Bars with asterisks represent group means with significant differences. Error bars represent 95% CI. Kruskal-Walli’s test was also conducted to determine if there were differences in the median values between groups that differed in their institute categories: ‘Academic Institute,’ ‘Secondary Healthcare Center,’ and ‘Tertiary Healthcare Center.’ Median values for the ‘Concern About Generic AEDs’ component were statistically significantly different between the different specialities, χ^2^(2) = 7.544, p = 0.023.

**Fig.3:**
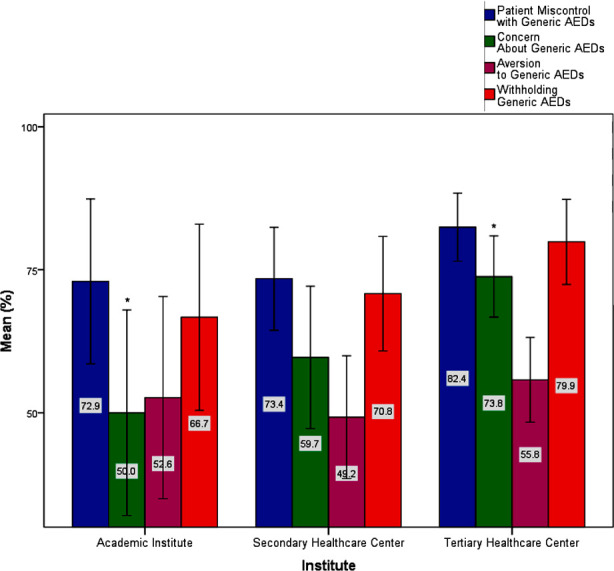
Assessment of neurologists’ perception of using Generic AEDs among different institute categories. Bars with asterisks represent group means with significant differences. Error bars represent 95% CI.

The post hoc analysis revealed statistically significant differences between the ‘Academic Institute’ (50.00) and the ‘Tertiary Healthcare Center’ (80.00) groups (p = 0.038). Pearson correlation analysis was made between the four neurologists’ perception assessment components. All four components were strongly and positively correlated with each other: the ‘Patient mis control with Generic AEDs’ component had statistically significant strong positive correlation with ‘Concern About Generic AEDs’ component, *r* (114) = 0.654, *p* < 0.0005; with ‘Aversion to Generic AEDs’ component, *r*(114) = 0.525, *p* < 0.0005; and ‘Withholding Generic AEDs’ component, *r*(114) = 0.595, *p* < 0.0005. ‘Concern About Generic AEDs’ component had a statistically significant strong positive correlation with the ‘Aversion to Generic AEDs’ component, r (114) = 0.538, p < 0.0005; and the ‘Withholding Generic AEDs’ component, *r* (114) = 0.459, *p* < 0.0005. Finally, the ‘Aversion to Generic AEDs’ component had a statistically significant positive correlation with the ‘Withholding Generic AEDs’ component, *r* (114) = 0.435, *p* < 0.0005.

Spearman correlation analysis revealed a positive correlation between years of experience and ‘Withholding Generic AEDs;’ *r* (114) = 0.243, *p* = 0.009. To confirm this correlation, Kruskal-Walli’s test was conducted to determine if there were differences in perception between groups that differed in their years of experience: the ‘< 5 years’, ‘5-10 years’, and ‘> 10 years’. Median values for the ‘Withholding Generic AEDs’ component were statistically significantly different between the various levels of experience, χ^2^(2) = 8.015, *p* = 0.018. The post hoc analysis revealed statistically significant differences between the low-experienced ‘< 5 years’ (66.50) and the high-experienced ‘> 10 years’ (100.00) groups (*p* = 0.014).

**Fig.4:**
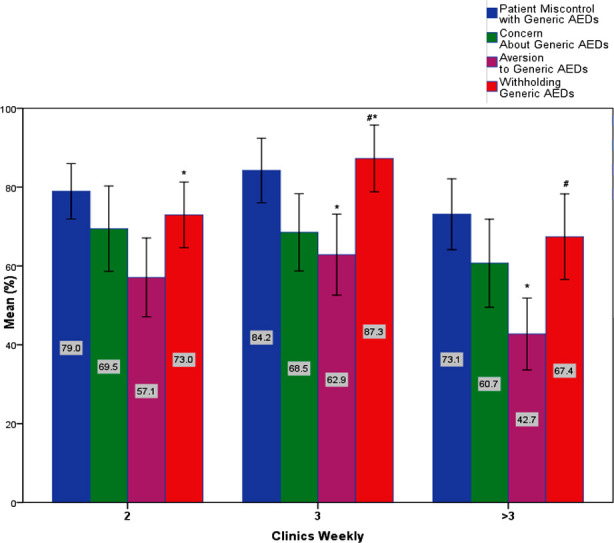
Assessment of neurologists’ perception of the use of Generic AEDs among different rates of weekly clinics. Bars with asterisks represent group means with significant differences. Error bars represent 95% CI.

Negative correlation between the number of clinics weekly and ‘Aversion to Generic AEDs’ *r_s_*(114) = -0.205, *p* = 0.029. To deeply investigate this pattern, the Kruskal-Wallis test was conducted to determine if there were differences in perception between groups that differed in their number of weekly clinics: the ‘2/ weeks’, ‘3/ weeks’, and ‘> 3/ weeks’. Median values for both the ‘Aversion to Generic AEDs’ component and the ‘Withholding Generic AEDs’ component were statistically significantly different between the various levels of experience, χ^2^(2) = 9.493, 9.664, *p* = 0.009, 0.008, respectively. Post hoc analysis defined the groups responsible for this significance: for ‘Aversion to Generic AEDs’ component the groups ‘3/ weeks’ (75.00) and ‘>3/ weeks’ (37.50) (*p* = 0.011); for ‘Withholding Generic AEDs’ component the groups ‘3/ weeks’ (100.00) and ‘>3/ weeks’ (66.50) (*p* = 0.015), and 2/ wk (66.50) and 3/ wk (100.00) (*p* = 0.024). Finally, the number of weekly patient visits had no significant correlation with any of the components. Fig.4 represents the differences among groups with different rates of weekly clinics.

## DISCUSSION

Epilepsy contributes to a significant disease burden in children and adolescents worldwide.^8^ We found that half of the neurologists in Saudi Arabia are concerned about substitution with generic AEDs. This alarming percentage raises the question regarding the efficacy of generic AEDs to control seizures in patients with epilepsy. Hence, the next component of this study was dedicated to estimating the neurologist’s perception of seizure miscontrol on generic AEDs. Of interest, two-thirds of neurologists who participated in this study “AGREE” that generic AEDs can lead to seizure miscontrol in patients with epilepsy. A statistically significant positive correlation existed between the ‘patient miscontrol with Generic AEDs’ component and the ‘Concern About Generic AEDs’ component. On the contrary, two-thirds “Disagree” or “Neutral” and only one-third “Agree” when it comes to aversion to generic AEDs; one key factor that may explain the responses seen in this component is cost efficacy. These findings suggest a mixed perception among neurologists regarding the use of generic AEDs for cost-saving purposes and the safety of substituting brand-name medications with generics.

Looking at previous studies to evaluate generic AEDs’ efficacy and cost-effectiveness, two randomized, double-masked studies were conducted in 2015-2016. No significant changes were recorded, whether in seizure frequency or adverse events. The results of the studies support the soundness of the FDA bioequivalence standards.^9^,^10^ In light of these findings, in 2016, the “American epilepsy society, AES, acknowledged that drug formulation substitution with FDA-approved generic products reduces cost without compromising efficacy.”^4^

However, both of these trials compared only generic lamotrigine to brand-name, and few previous studies conducted earlier in 2015^11^,^12^ suggested statistically higher overall healthcare costs when substituting branded antiepileptics with the generic alternative. This price increase was connected to “ranges of bioequivalence authorized for generic formulations do not offer the same results regarding effectiveness and safety as those obtained by brand-name drugs.”^11^ A critical review study in 2018 concluded that there is minimal risk from switching to generic AEDs and significant cost-effectiveness. They suggest, “When patients are administered a new AED, due to lower costs, a generic should be used if available.”^13^,^14^.

Generic substitution of antiepileptic drugs remains a controversial area without a clear consensus to guide clinicians. These controversies are reflected in neurologists’ perceptions and practices in our study, despite the results of controlled clinical trials. Implementing stricter regulations regarding the use and switching to generic AEDs is crucial to shaping the perception and practice of substituting generic AEDs in Saudi Arabia. Sixty-six per cent of neurologists participated in “AGREE” to withhold generic AEDs and seek physician approval before switching to generic AEDs. Withholding generic AEDs would provide better opportunities for patients’ education, which can improve physicians’ and patients’ perceptions and practices regarding the’ control of seizures by generic AEDs.^15^ Epilepsy Foundation highly advises physician approval before switching to generic AEDs.^16^

Observing statistically significant variance and correlations in our study, we believe that addressing the following factors in designing future studies will help better evaluate and estimate the substitution of brand medications with generic alternatives. Female neurologists are more concerned than males, and pediatric epileptologists discourage switching to generic AEDs compared to pediatric general neurologists and adult neurologists, giving this age group special consideration when switching to generic AEDs. Of interest is that tertiary health care centers are significantly more concerned regarding generic AEDs than academic centers. Also, physicians with more experience (above 10 years) are more likely to withhold generic AEDs; these show the significance of patient and physician factors in optimizing perception and practice towards switching to generic AEDs.

### Study strengths and limitations:

In our study, we relied on physician reports of health information, which can be biased by previous incidents or personal preference. Although we cannot predict whether a bias exists, having an actual image of perception and practice is what we target; in the real world, this bias can direct the perception and even the practices. One hundred fourteen certified neurologists in Saudi Arabia participated in this research; this sample size is a good representative of neurologist’s perceptions and practices in Saudi Arabia. Ninety-six per cent of participants are from Urban areas clustered in Central and western regions; we encourage future studies to expand more geographically.

## CONCLUSION

It was determined that fifty per cent of Saudi Arabian neurologist express apprehension regarding the replacement of brand-name AEDs with generic alternatives. Neurologists have varied perspectives concerning using generic AEDs for cost reduction and the safety implications of substituting established brand-name medications with their generic counterparts. We advocate for the documentation of every occurrence of breakthrough seizures in patients who are being treated with generic AEDs.

### Author’s contribution:

**BNA:** Research design, supervision, writing and editing, responsible and accountable for the accuracy and integrity of the work.

**RNA, RSA, HE and MHB:** literature review, data collection and analysis.

All authors have read and approved the final version of the manuscript.
